# Spillover effect analysis of home-purchase limit policy on housing prices in large and medium-sized cities: Evidence from China

**DOI:** 10.1371/journal.pone.0280235

**Published:** 2023-01-10

**Authors:** Guancen Wu, Wenjing Guo, Xing Niu

**Affiliations:** 1 School of Management, Shanghai University, Shanghai, China; 2 School of Social and Public Administration, East China University of Science and Technology, Shanghai, China; Massey University - Albany Campus: Massey University - Auckland Campus, NEW ZEALAND

## Abstract

Home-purchase limit is a unique administrative housing policy of China and has non-negligible influences on the housing price. The objective of this study is to analyze the spillover effect of home-purchase limit policy on housing prices in 35 large and medium-sized cities. The panel data of these cities and the spatial Durbin model are employed in this study. The results indicate that the spillover effect of home-purchase limit policy is positive and significant in all of 35 cities. However, when we categorize these cities into high-risk, medium-risk, and low-risk based on housing price characteristics, the spillover effect of home-purchase limit policy is different. It is not significant in high-risk cities, is negatively significant in medium-risk cities, and is positively significant in low-risk cities. This paper suggests that local governments can pay more attention to the precise design and implementation of home-purchase limit policy, and maintain policy continuity to avoid further spillover fluctuations in housing prices.

## Introduction

With the development of economic integration, housing prices in various cities are starting to influence each other. This phenomenon of spatial transmission of housing prices can be called the “spillover effect”. This has been confirmed by studies not only in the U.K. [1] but in the U.S. [2], Korea [3], and many other countries as well. Many studies have shown that urban housing prices in China also have an noticeable spillover effect [4–6]. Rising housing prices in megacities have caused other cities to follow irrationally. The housing price spillover has exacerbated the imbalance in the housing market, resulting in national socioeconomic risks [7].

In general, housing policies can regulate the housing market. Since 2003, the Chinese central government has introduced a series of policies to curb the rapid rise in housing prices. Only there are two short periods of exceptions when home purchases were encouraged. One happened in 2008 after the global financial crisis. China’s housing market quickly recovered from this financial crisis and housing prices kept rising uncontrollably [8]. On April 17, 2010, the home-purchase limit policy was issued by the State Council of the People’s Republic of China, which is as a response to the prohibitive housing prices.

Different from conventional housing policy tools, the home-purchase limit policy restricts the number of houses that a given resident household can buy in a city. For example, resident household with local *hukou* was directly prohibited from buying more than two housing units, and resident household without local *hukou* who had been working and paying social security or income tax for some straight years could not buy more than one housing unit. In China, *hukou* is a system of household registration that can determine access to housing, education, medical treatment, and other social welfare rights.

Due to differences in the housing market, local governments have much discretion in implementing the home-purchase limit policy. Beijing released its first local version of home-purchase limit policy in 2010, other megacities such as Shanghai, Guangzhou, and Shenzhen also implemented one after the other. But many second-tier and third-tier cities loosened their home-purchase limit policy in 2014 due to the depressing domestic macro environment. The other period which encouraged home purchases occurred in 2015 when the government intended to reduce the number of unsold homes. Subsequently, the home-purchase limit policy was tightened again and even reinforced in 2016.

When Shanghai extended the social insurance or personal income tax payment period from two to five years in March 2016, the chain housing price indexes were 103.1 in April, 101.9 in May, and 102 in June. The chain housing price indexes of the neighboring city Nanjing, rose respectively to 104.1, 103.9, and 103.8. The chain housing price indexes respectively were 102.3, 103, and 102.5 in the neighboring city, Hangzhou. But in February, the chain housing price indexes respectively were 102.6 in Nanjing and 101.3 in Hangzhou. These two cities have less stringent restrictions on home purchases than Shanghai, and the chain housing price index has increased even more.

The main purpose of this study is to examine whether housing prices of these large and medium-sized cities also are affected by changes in external cities’ home-purchase limit policy. This study also hopes to explore whether there is heterogeneity in the spillover effects of home-purchase limit policy on housing prices in cities with various housing markets. The conclusions can be more beneficial for analyzing fluctuations in the national housing market in China, and be instructive for local governments to formulate a home-purchase limit policy. Our research also is helpful to other countries and regions interested in providing sustainable and stable housing policies.

The following section 2 provides related literature review. Section 3 presents the methodology and data. In section 4, we examine the spatial correlation of housing markets in 35 large and medium-sized cities, use spatial econometric model to analyze the spatial spillover effect of home-purchase limit policy, investigate the heterogeneity of the spillover effect in cities with different housing market types and discusses the regression results. Finally, we conclude this paper and propose some policy suggestions in Section 5.

## Literature review

### Existence of spillover effect

Early work on the spillover effects of housing prices began in the U.K.. Macdonald and Taylor [9] analyzed the panel data on housing prices in different regions of the UK for the first time and found co-integration relationships between several areas of the UK. Alexander and Barrow [10] also verified the existence of the spillover effect. Cook and Thomas [11] pointed out that the ripple effect of housing prices in the south of England is more extensive than in other regions of the UK.

More and more studies from other countries have shown the existence of spatial spillover effects on housing prices. Clapp and Tirtiroglu [12] argued that housing prices between neighboring cities in the U.S. have a spatial spillover effect. But Pollakowski and Ray [2] suggested that only the Greater New York metropolitan has housing price spillover. Berg [13] and Oikarinen [14] found housing spillover effects in Sweden and Finland, respectively. Greg et al. [15] identified the existence of housing spillover among the capital cities of states in Australia.

China’s housing prices across cities have increasingly connected [4]. Weng and Gong [16] indicated strong spillovers among ten cities with close geographic and economic proximities, including first-tier and second-tier cities in China. Yu [17] also found that Beijing and Shanghai have comparatively significant spillovers of housing prices, while spillovers in central and western cities are insignificant. Tsai and Chiang [18] argued that Beijing first exhibits housing market exuberance, which is then transmitted to second-tier cities.

### Influence factors and heterogeneity of spillover effects

Many scholars studied the factors that influence the spillover effect on housing prices. Meen [19] indicated that population, wealth, and investment are the main factors affecting the spillover effect of housing prices. Traditional time-series model and different vector autoregression models have been used to recognize the source of housing price spillover effects in many countries [4, 20, 21]. Lu et al. [5] found that inter-regional migration and capital flow have positive impacts on the spatial spillovers of housing prices. Yang et al. [22] showed that population, GDP, and secondary education are significant determinants of housing price spillovers. Kuethe and Pede [23] further illustrated how one region’s actual income and unemployment affect other regions’ housing prices. It can be seen that the influential factors of the spillover effect of housing prices are very complex [24].

With the advancement of spatial econometrics, Spatial Weight Matrix was introduced into traditional econometrics models, which enriched the study of the spillover effect of housing price [25]. Holly et al. [26] constructed the Spatial Panel Data Model of 49 states in the United States during 1975–2003 and indicated a significant housing price spillover effect in neighboring states. Cohen et al. [27] showed that the degrees of housing price spillover effect after the 2008 financial crisis were more rapid.

Spatial connections of housing prices did not only rely on geographical adjacency relations. Bhattacharjee and Jensen-Butler [28] found that spatial diffusion was substantially influenced by traffic links than distance. The study results of van Dijk et al. [29] in 76 cities in Holland showed that the group with a higher housing price growth rate exhibited faster and more robust responses to changes in GDP compared to their counterparts. Zhang et al. [30] used a spatial panel model with geographical distance and economic distance weight matrices to analyze the housing price spatial effects of Yangtze Delta urban agglomeration in China.

Some researchers have conducted an in-depth heterogeneity analysis of housing price spillover effects. Lan et al. [31] adopted the gravity model and time-space model with 100 cities data in China. They found that the degree of spillover effect between cities was correlated to economic foundations and the grade of cities in general. In urban agglomerations with a higher degree of networked structure, the spillover effect of the central city was more dominant. Coën et al. [32] used a spatial panel vector autoregressive model to analyze and discuss the ripple effects across housing submarkets. Results found that cities in the same cluster were interconnected rather than alone, and the contribution of each variable to housing prices varied by submarket. Alberto and Jun [33] suggested there were more stronger spillover effects from the core regions in the housing market across 12 UK regions. Zhang et al. [34] investigate the ripple effect of house prices among 35 metropolitans in China and found more stronger ripple effects in the region of a higher level of economic development. Brady [35] analyzed the spillover effect of regional housing prices between different sub-periods.

### Home-purchase limit policy and housing price

Policies are validated by many studies as a significant factor affecting housing prices, like economic policy uncertainty [36], rent control [37], forced sale [38], land policy [39], monetary policy [40], and so on. Lu et al. [5] have shown that the remarkable peaks of spatial spillover parameters coincided well with some governmental interventions in housing prices of 70 large and median cities. Lan et al. [31] believed that suitable policies for each city’s circumstances could prevent the overall spillover of housing prices.

The home-purchase limit policy is a unique policy tool in China, and implementation details vary from city to city and even from year to year. Different research designs, as well as different study periods and even different sample cities, may lead to different conclusions.

Yan et al. [41] focused on the local impact of home-purchase limit policy on housing prices in a city. This study used a quasi-experimental test with yearly data on 287 Chinese cities from 2007 to 2013 and found that the home-purchase limit policy has led to a reduction in housing prices on average. The more stringent the policy, the more controlled housing prices are. But Jia et al. [42] investigated the effects of the home-purchase limits based on the microdata of resale housing transactions between January 2008 and December 2011 in Guangzhou city and found that localized measures positively affect the prices of resale homes. Similar findings have been found in a large number of Chinese journals. Deng et al. [43] found that the home-purchase limit policy did not show a restraint effect on housing prices in cities with “excessively rapid rises in housing prices”. Dong and Wang [44] believe that the home-purchase limit policy was affected by the inadequate implementation of local policy and exacerbated housing market volatility.

Some scholars suggested that home-purchase limit policy was not always effective in suppressing housing prices. Huang et al. [45] found that the effect of home-purchase limit policy in pilot cities was initially more effective, but tended to lose effectiveness after a long period of time. Fang [46] found that the second round of home-purchase limit policy was weaker than the effect of the first round. Li et al. [47] indicated that home-purchase limit policy only reduced the growth rate of housing prices, but it could not change the long-term tendency of housing prices. When the restriction slowed down, it would bring about a retaliatory rebound.

Most of the scholars have found heterogeneity in home-purchase limit policy. Mi and Liu [48] suggested that home-purchase limit policy reduces housing prices and has a heterogeneous effect among cities. The higher the proportion of real estate investment in GDP, the greater the impact of a home-purchase limit policy. Yu [17] noticed that regional home-purchase limit might restrain external housing purchase funding from flowing to the first-tier and eastern cities in a short time. Sun et al. [8] found that the effects of the home-purchase limit policy were larger in price and smaller in quantity, especially in submarkets where the housing supply was less elastic. Not only are there heterogeneities in home-purchase limit policie in various cities, but Lia et al. [49] also suggested that the effect of the home-purchase limit policy on housing prices had spatial heterogeneity within the same city. The result is based on second-hand housing transaction data from Langfang City, which is adjacent to Beijing, the capital of China.

In particular, Cao [50] believed that the growing intensity of purchase restrictions in neighboring cities would promote the power of home-purchase limit in local government. This can also lead to uncertainty in the housing market [42].

### Summary

The main purpose of this paper is to examine whether a city’s home-purchase limit policy has a spillover effect on the housing prices of other cities in large and medium-sized cities in China. Meanwhile, this paper also tries to explore the heterogeneity of the spillover effects of home-purchase limit policy in cities with different housing markets. The Spatial Dupin model is employed, which has been maturely applied in analyzing the spatial spillover effect.

Below are the major contributions of our study. First, previous literature on housing price spillovers focused more on the spillovers from housing prices themselves. Existing studies on the factors influencing the housing prices spillover also mainly considered supply and demand, the level of economic development, and so on. Our study looks at changes in home-purchase limit policy as a factor affecting housing price spillovers. This can be as a complement to the housing price spillovers study and policy spillovers study. Second, previous literature focused mainly on the impact of the city’s home-purchase limit policy on local housing prices. Our study further examines the impact of home-purchase limit policy on housing prices in other cities. Most early works examined the effects of the first round of home-purchase limit policy after 2010. These analyses were generally based on the comparison of the effect with the home-purchase limit policy to that without home-purchase limit policy. This paper considers the cancellation and re-tightening of home-purchase limit policy in individual cities and quantifies the strength of home-purchase limit policy. The findings of this analysis are relatively more precise. Third, unlike some studies that focus on the short-term impacts of home-purchase limit policy on housing prices, this paper is more interested in examining the effects of changes in a city’s home-purchase limit policy on average annual housing prices in other cities. It is more appropriate reference for local governments or central government to consider relaxing or tightening home-purchase limit policy prudently. Fourth, some studies focused more on the housing price spillover from large cities to neighboring small cities. This study focuses more on large and medium-sized cities.

Furthermore, the paper classifies the housing market risk to explore the heterogeneity of home-purchase limit policy according to the housing price and growth rate. In this way, it is easier to analyze the spillover mechanism of the home-purchase limit policy. It is helpful for local governments and the central government to prevent housing market risks from a macro view. Local government also can design better housing policies for each city.

## Research design

### Spillover mechanism of home-purchase limit policy

According to the policy design objective, one city’s home-purchase limit policy would not fundamentally increase the actual supply of housing units, but it would limit the housing demand. In addition to curbing speculation on purchasing more home units, the rigid demand for housing of some households without local *hukou* will also be restricted. That is, home-purchase limit policy restricts options for groups who want to enter the city to work and live, including many young graduates from university. They are renting or leaving this city. In the case of a partial reduction in demand and constant supply, this city’s housing market may experience a fall in prices or a decline in the upward trend. That is the direct impact of a city’s home-purchase limit policy on its housing prices.

Of course, it should also be noted that home-purchase limit policy primarily intended to reduce speculative demand. However, it is difficult to play a fundamental role in the decline in housing prices, particularly in cities where net population entries and housing prices are extremely high.

People who have already chosen to leave this city may move to other cities with less restrictive home-purchase limit policy or where they can be satisfied. This part of housing demand creates an additional external shock on housing prices in other cities. It’s the spillover effect of the city’s home-purchase limit policy on housing prices in other cities.

Furthermore, this study also estimates that home-purchase limit policy spillover effect will link to the cities’ housing market conditions, including the basis of housing prices, the growth trends in housing prices, and the implementation intensity of their home-purchase limit policy.

If a city’s basic housing prices are higher and growing faster, the city’s housing market is risker. The city’s home-purchase limit policy may be stricter, and housing prices are more influenced by its own housing supply and demand. It is not easy to respond to the external demand shock caused by the home-purchase limit policy in other cities. However, if the basic housing prices in a city are low, and the growth rates are not high, then the city’s housing market is less risky. As home-purchase limit policies tighten in other cities, this city is more likely to absorb excess housing demand, which leads to a higher housing price in this city. If the home-purchase limit policy of a city is relatively stringent, the housing construction is not sufficiently profitable. It may lead real estate developers to develop housing projects in other cities. At this time, housing prices in other cities could decline in due to increased supply. This applies to the cities where the demand for housing is not growing.

### Spatial Durbin model

Some findings on house price spillover were based on time-series data and the VAR model. The dependence on other factors which affect the housing price spillover could be disregarded. LeSage and Pace [51] indicated that if it is necessary to consider both explained variables with spatial lag effect and their influence on the explained variable, the spatial econometric models can be used. According to different spatial dependencies, main spatial model types include spatial lag, spatial error, and spatial Durbin model. The spatial Durbin model is widely used to analyze spillover issues about housing prices [25, 27, 28, 52, 53].

Because this paper focuses on the spillover effect of home-purchase limit policy on neighboring cities’ house prices, the spatial Durbin model (SDM) fits well with the purpose of the study and reality. Of course, this paper also constructs a spatial lag model (SLM) and spatial error model (SEM), and illustrates the selection of spatial econometric models and relevant tests.

The spatial Durbin model used in this paper can be specified as follows:

$$Y_{it}=\rho WY_{it}+X_{it}\beta +{Wpolicy}_{it}\delta +u_{i}+v_{t}+\varepsilon_{it}$$ (1)

Where Y_it_ is the *n*×1 vector of dependent variable, the subscript *i* represents the city, and the *t* represents the year. ρ is the spatial autoregressive parameter, *W* denotes the *n*×*n* spatial weight matrix. *X*_*it*_ is the *n*×*k* matrix of main explanatory variable *Policy*_*it*_ and other control variables. In this study, *policy*_*it*_ is defined by a dummy variable that varies with the strictness of home-purchase limit policy, and the dependent variable of housing price and other explanatory variables are in the form of natural logarithm to reduce possible heteroscedasticity problems. *β* is the *k*×1 vector of regression parameter. *δ* is the regression coefficient to be estimated. *u*_*i*_、*v*_*t*_ and *ε*_*it*_ are space effect, time effect, and perturbation vector, respectively.

Since the δ in the model may ignore the interaction information between neighboring cities, the spatial spillover effect of explanatory variables cannot be truly represented. LeSage and Pace [54] define the matrix ${{(I}_{n}-\rho W)}^{-1}({I_{n}\beta }_{x}+W\delta )$, where *I*_*n*_ is an *n*×*n* unit matrix, *β*_*x*_ is the regression coefficient of *X*. For the main explanatory variable *policy*_*it*_ of this paper, the matrix is ${{(I}_{n}-\rho W)}^{-1}({I_{n}\beta }_{policy}+W\delta )$, it can be expressed as follow:

$${{(I}_{n}-\rho W)}^{-1}({I_{n}\beta }_{policy}+W\delta )={{(I}_{n}-\rho W)}^{-1}\left[\begin{array}{ccc} \beta_{policy} & w_{12}\delta_{t}\cdots & w_{1n}\delta_{t}\\ \vdots & \ddots & \vdots \\ w_{n1}\delta_{t} & w_{n2}\delta_{t}\cdots & \beta_{policy} \end{array}\right]$$ (2)

The average of the diagonal elements represents direct impact, while the mean row sum of off-diagonal elements denotes indirect impact (spillover effect). The average direct impact plus the average indirect impact equals the average total impact.

### Spatial weights matrix

The spatial weight matrix is the main form to measure the spatial interdependence degree between observation units. To some extent, the setting of spatial weights will also affect the spatial analysis results. Therefore, the spatial weight matrix should be based on the actual situation and research purpose. So far, scholars have commonly used distances including adjacency distance and geographic distance. Considering the price spillover from one city to other cities will decrease as the distance increases. The spatial weight matrix is specified as follows:

$$w_{ij}=\left\{ \begin{array}{c} 1/d_{ij},i\neq j\\ 0,i=j \end{array}\right.$$ (3)

where *w*_*ij*_ reflects the element in the *n*×*n* vector spatial weight matrix (*W*), *i* and *j* stand for the city *i* and city *j*; *d*_*ij*_ denotes the straight-line geographic distance between city *i* and city *j*. Generally, *W* is row-normalized, and is not endogenous.

### Variable choice and descriptive statistics

#### Home-purchase limit policy

Home-purchase limit policy is the main explanatory variable and is a measure to control speculative demand by limiting the number of home-purchase. The 2010 “Ten New National Rules” marked the beginning of the nationwide home-purchase limit policy. Local home-purchase limit policies were implemented in 46 cities, and housing prices were implemented and effectively controlled. But due to the pressure of the housing stock increase, Hohhot city eased its limit policy on home purchases in June 2014. Subsequently, most cities canceled their home-purchase limit policy one after another.

After the end of the first round of home-purchase limit policy, housing prices in various cities began to increase irrationally again. In 2016, some cities reintroduced or revised their home-purchase limit policies. Until now, individual cities continue to adjust and modify their home-purchase limit policies based on local housing market conditions.

Generally, the home-purchase limit policy states that local residents of the city who already own a home can only purchase new housing, and non-residents of the city are required to pay urban social insurance or personal income tax for a number of years before the date of purchase. This paper argues that the more years of paying urban social insurance or personal income tax, the stricter the home-purchase limit policy. A dummy variable is introduced as a proxy variable for the home-purchase limit policy. If one city does not implement or cancel the home-purchase limit policy, the dummy variable is set to 0. If one city implements home-purchase limit policy for local residents who already own a home and non-residents who have no home, the dummy variable is set to 1. When non-residents need to pay an additional year of urban social insurance or personal income tax, the value of the dummy variable is plus one. The dummy variable is at most 6 because non-residents need to pay urban social insurance or personal income tax for a maximum of 5 years. The specific values are described in Table 1.

**Table 1:** Specific description of the indicators of home-purchase limit policy.

Indicator	Qualification to purchase a new home
**Home-purchase limit policy**	0: no home-purchase limit policy 1: local residents who already own a home and non-residents who have no home 2: local residents who already own a home, and non-residents who have no home and pay urban social insurance or personal income tax for 1 year 3: local residents who already own a home, and non-residents who have no home and pay urban social insurance or personal income tax for 2 year 4: local residents who already own a home, and non-residents who have no home and pay urban social insurance or personal income tax for 3 year 5: local residents who already own a home, and non-residents who have no home and pay urban social insurance or personal income tax for 4 year 6: local residents who already own a home, and non-residents who have no home and pay urban social insurance or personal income tax for 5 year

#### Dependent variable and other variables

Housing prices are selected as the dependent variable. According to the existing research [23, 30, 31], population and GDP are important influence factors on housing prices in the city itself and in other cities. Other variables like education [22], and income [17, 21] were selected for study purposes. So this study also includes population and GDP, which are considered to improve the accuracy of the study results.

#### Data source and descriptive statistics

The sample of this study included 35 large and medium-sized cities. These cities are all provincial capitals or economically developed cities throughout China. They have different characteristics of housing supply as well as market demand. In addition, these cities had home-purchase limit policies with different strengths since 2010 and have undergone the changes in different directions. Therefore, the data from these cities can meet the research objective and provide an important reference for government policymaking.

In this study, we create an annual dataset. It is because we do not want to analyze the fluctuation of short-term housing prices caused by home-purchase limit policy. Instead, we would like to observe a stabilizing spillover effect of home-purchase limit policy on housing prices over a long period. Another reason is that the population indicator and GDP indicator are available at an annual frequency. Data for these two variables are obtained from the 2010 to 2019 China City Statistical Yearbook, the statistical bulletin of each city. The unit of population indicator is ten thousand, and the unit of GDP is one hundred million yuan.

The housing price indicator used is the annual average price obtained from monthly data in the WIND database. The WIND database includes various financial market data and China’s macro-industry, even the monthly housing price. Data on housing prices come from existing residential units, which are more relevant to this study and cannot be provided in the National Statistical Yearbook. The unit of housing price indicator is yuan/m^2^.

The geographical distances between cities are calculated by ArcGIS 10.2 using Euclidean distances based on the projected coordinates converted from city latitude and longitude coordinates.

As shown in Table 2, there are significant differences in the data of 35 cities in China. The gap between the maximum and minimum values of each indicator is relatively significant. Most indicators fluctuate more obviously. This shows a great imbalance in the development of the housing market among the 35 cities. There is a total of 350 sets of observed data.

**Table 2:** Definitions of variables and descriptive statistics.

Indicator	Abbreviation	Definition	Min	Max	Mean	Std. dev.
**Housing prices**	hp	Urban existing residential housing prices	4585	54313	12294.84	9116.22
**Home-purchase limit policy**	policy	Strictness of policy	0	6	1.59	1.56
**Population**	pop	Population at the Year-end	182	3416	885.03	598.50
**GDP**	gdp	Gross Domestic Product	595	38155	7759.47	6604.13

**Fig 1:**
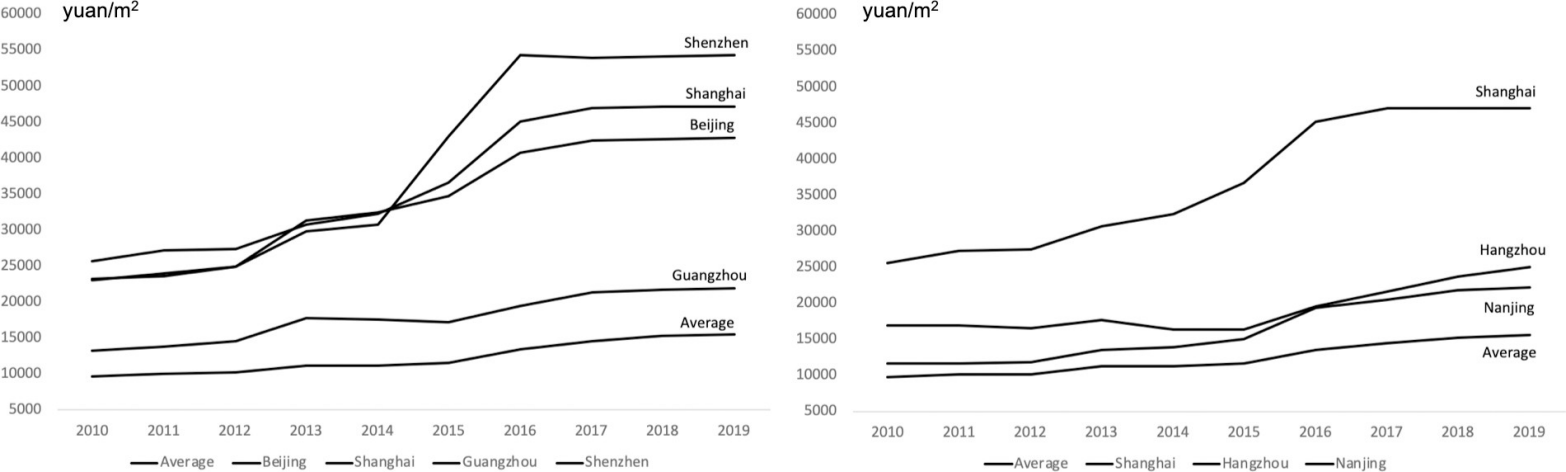
Average housing prices and housing prices in major cities.

Fig 1A demonstrates the trends in average housing prices of all sample cities and housing prices of four first-tier cities. The four cities are Beijing, Shanghai, Shenzhen, and Guangzhou, and they are not very close in geographical terms. The average housing price of all sample cities has risen steadily over time. It can be seen that housing prices in Shanghai and Shenzhen were somewhat controlled as home-purchase limit policies were tightened in 2016. However, housing prices in Guangzhou were still rising, and the city’s home-purchase limit policy was being tightened in 2017.

Fig 1B demonstrates the housing price movements in Shanghai and the neighboring cities of Hangzhou and Nanjing. Nanjing’s home-purchase limit policy has remained relatively less strict, Hangzhou’s home-purchase limit policy was canceled once in 2014 and tightened again in 2017. And housing prices in these two cities behaved differently in 2016 when Shanghai’s home-purchase limit was tightened. Housing prices in Hangzhou rose significantly in 2016 and 2017, while housing prices in Nanjing only increased dramatically in 2016 and then returned to the previous stable trend.

## Empirical analysis and discussion

### Spatial correlation analysis

To test whether housing price has a spatial effect among large and medium-sized cities, Table 3 shows Moran’s I result from 2010 to 2019.

**Table 3:** Moran’s I of housing prices among large and medium-sized cities.

Year	2010	2011	2012	2013	2014	2015	2016	2017	2018	2019
**Moran’s I**	0.133	0.133	0.127	0.122	0.117	0.108	0.119	0.127	0.127	0.127

The result of Moran’s I is greater than 0 for all years. It can be shown there is a significant positive correlation between housing prices. Since 2011, this positive spatial correlation of housing prices gradually began to weaken. In 2015, the positive spatial correlation of housing prices began to increase. This is also consistent with the relaxation and tightening of the home-purchase limit policy. As found in the literature [5], the spatial spillover parameters coincide with some government intervention policies. But the specific spatial spillover effect of the home-purchase limit policy on housing prices needs to be further studied with the help of spatial econometric models.

### Test of model applicability

Selecting a suitable model is an essential step in spatial econometric analysis. The likelihood ratio (LR) test and Wald test are used to determine whether the SDM is more suitable than spatial lag model (SLM) or spatial error model (SEM). The results show that the Wald and LR tests rejected the null hypothesis of simplifying the SDM into SLM or SEM.

Furthermore, based on the results of the Hausman test, a fixed-effect model was selected for the estimation. All the results can be seen in Table 4.

**Table 4:** Regression results of different tests.

Model	Spatial lag model	Spatial error model	Spatial Durbin model
**WALD test**	23.61***	26.43***	
	(0.000)	(0.000)	
**LR test**	28.84***	28.17***	
	(0.000)	(0.000)	
**Hausman test**			32.35***
			(0.000)

Note: ()represent the P value

*, **, *** indicate statistical significance at 10%, 5%, 1% level, respectively.

### Basis regression results

The estimation results of the common panel model and SDM are shown in Table 5. The coefficient of policy in SDM is lower than in the non-spatial effect model. The coefficient of the Wpolicy is 0.182 with a statistical significance of 1%, which means the policy has a positive space spillover effect. The common panel model underestimates the impact of policies on housing prices of local cities and other large and medium-sized cities.

**Table 5:** Comparison of empirical results of housing price spatial panel model.

Variables	Non-spatial effect	SDM
**policy**	0.054 ***	0.051***
**lnpop**	0.199	0.085
**lngdp**	0.064	0.094
**Wpolicy**	/	0.182***
**Wlnpop**	/	0.548
**Wlngdp**	/	-0.791***
**σ** ^**2**^	/	.006***
**Spatial rho**	/	-0.383
**Log-likelihood**	/	395.995
**R** ^**2**^	0.455	0.168

Note

*, **, *** indicate statistical significance at 10%, 5%, 1% level, respectively.

Table 6 shows the estimated results of the direct effect, spillover effect, and total effect. The direct effect measures the impact of each factor on local housing prices, and the spillover effect measures the impact of each factor on housing prices in other large and medium-sized cities. The total effect is equal to the sum of the direct and spillover effect. The results show that there are significant direct effects and spillover effects of both policy and GDP. The spillover effect efficiency of policy is 0.123, and the direct effects efficiency of policy is only 0.049. This shows that the home-purchase limit policy impacts housing prices not only in the city itself but especially in other large and medium-sized cities. It can be seen that with every 1% increase in GDP, the housing prices of the local cities will increase by 0.107%, and the housing prices of other cities will decrease by 0.628%. That is, GDP has a significant positive direct effect, which is consistent with most of the findings in the existing literature. But GDP has a significant negative spillover effect. This means that when a city’s GDP increases, the housing prices in other cities decrease. This finding does not appear in other studies. Due to the different research designs, some previous studies only mentioned that GDP would increase the external spillover of housing prices in a certain city.

**Table 6:** Empirical results of decomposition of spatial effect based on the asymmetric weight matrix.

Variables	Direct effect	Spillover effect	Total effect
**policy**	0.049***	0.123***	0.172***
**lnpop**	0.078	0.409	0.487
**lngdp**	0.107*	-0.628***	-0.520**

Note

*, **, *** indicate statistical significance at 10%, 5%, 1% level, respectively.

### Estimates results with consideration of housing market

Considering the heterogeneity of the spillover effect in the housing market, this paper divided large and medium-sized cities with different housing markets. This paper used the housing prices, the growth rate of housing prices, and the variation coefficient of housing prices in 35 cities from 2010 to 2019 to perform a cluster analysis. The 35 cities were divided into three market types. Among them, Beijing, Shanghai, and Shenzhen are high-risk housing markets with high housing prices, high growth, and high variation coefficient. The specific list of medium-risk cities and lower-risk cities is shown in the following Table 7.

**Table 7:** Attributes and classifications of urban housing market of cities.

Urban area	Housing price	Growth rate	Variation coefficient	Specific cities	Degree of home-purchase limit policy
**High-risk**	36584.44	8.44%	0.2668	Beijing, Shanghai, Shenzhen,	4.27
**Medium-risk**	15437.19	4.65%	0.1629	Tianjin, Dalian, Nanjing, Hangzhou, Ningbo, Fuzhou, Xiamen, Guangzhou, Haikou,	2.00
**Low-risk**	7895.46	4.75%	0.1413	Qingdao, Nanchang, Zhengzhou, Hefei, Wuhan, Changsha, Chongqing, Shijiazhuang, Urumqi, Taiyuan, Jinan, Harbin, Shenyang, Changchun, Hohhot, Nanning, Chengdu, Guiyang, Kunming, Xi’an, Lanzhou, Xining, Yinchuan	1.07

The low-risk cities have low housing prices, low growth, and low variation coefficient. While medium-risk cities have higher housing prices than low-risk cities. The strength of the home-purchase limit policy is also the largest in high-risk cities and the lowest in lower-risk cities. In particular, most medium-risk cities and low-risk cities canceled their home-purchase limit policy in 2014 or 2015, and then some cities reimplemented it.

According to the above classification standard of urban housing market heterogeneity, the model estimation results of the housing spillover effect under high-risk, medium-risk, and low-risk housing markets are shown in Table 8.

**Table 8:** Empirical results of decomposition of spatial effect based on the asymmetric weight matrix.

Variables	Direct effect			Spillover effect		
	High-risk	Medium-risk	Low-risk	High-risk	Medium-risk	Low-risk
**policy**	0.043**	0.066***	0.025***	0.062	-0.107***	0.209**
**lnpop**	0.099	-0.082	0.303**	0.150	0.223	2.213
**lngdp**	1.623***	0.202	0.057	0.841	-0.031	-1.166***

Note

*, **, *** indicate statistical significance at 10%, 5%, 1% level, respectively.

The direct effect of the policy is still positive across all regions. Efficient of policy is significant at the 5% level in high-risk areas and is significant at the 1% level in medium-risk and low-risk areas. The coefficient of 0.066 for medium-risk areas is higher than 0.042 for high-risk areas and 0.025 for low-risk areas. However, it is worth noting that the spillover effect of the policy becomes insignificant for high-risk areas and is negatively significant at -0.107 at the 1% level for medium-risk areas.

The direct effect of GDP is positive only in high-risk areas. The spillover effect is -1.166 at the 1% significance level in the low-risk areas.

Another concern is the population size, where each 1% increase in the low-risk area can result in a housing price growth of 0.303%.

## Discussion

The result of the home-purchase limit policy slightly promoting the rise in local housing prices is still consistent with the reality of the housing market in China. The original intention of the home-purchase limit policy was not to reduce housing prices but to suppress speculative demand and meet rigid demand. These have also been demonstrated theoretically and empirically in literature [55, 56].

We also can see that the spillover effect is pronounced in 35 large and medium-sized cities and is greater than the boost effect on local housing prices. This can be explained by the differentiated home-purchase limit policies and the imbalance of public goods in these cities. When the home-purchase limit policy changes, people move from one city to another with different public resources. This changes the supply and demand of the housing market and results in fluctuating housing prices in these cities. This is a valuable supplement to existing literature, including policy spillover or housing price spillover. That is, from the perspective of all large and medium-sized cities, rising demands for housing in other cities with relatively less restrictive home-purchase limit policy have led to an extra rise in urban housing prices.

This paper also has many new findings through the analysis of heterogeneity. The direct impacts and indirect spillover effects of home-purchase limit policy are different in cities with different housing markets risks. With a more restrictive home-purchase limit policy, people may perceive higher future housing values in medium-risk cities, causing an increase in housing prices. In high-risk cities, this possibility exists as well. But perhaps the combination of other policies, such as restrictions on sale prices and complementary policies on loan interest rate adjustments, has more effectively controlled the increase in local urban housing prices. Medium-risk cities would like to increase property taxes for urban development without adding other additional regulations. The home-purchase limit policy of low-risk cities has a more minor impact on local housing prices because local demand remains relatively inadequate in the overall national housing market.

GDP growth in high-risk cities has a significant impact on the rise of local housing prices, which is consistent with previous studies in which GDP drives home price inflation. It also suggests that the housing market is more independent in high-risk cities. The results of population growth in low-risk cities leading to higher housing prices indicate the demand-oriented housing market in low-risk cities.

The spillover effect of the home-purchase limit policy is not significant in high-risk cities, which also indicates that the rising housing prices is mainly caused by local supply and demand.

In order to strengthen competition for talent among cities, each medium-risk city implemented its own unique home-purchase limit policy with different intensities. A number of cities, such as Dalian, Nanjing, Hangzhou, Ningbo, and Fuzhou, have canceled home-purchase limit policies at various times and have subsequently re-established them. In general, the degree of home-purchase limit policy in most medium-risk cities is no more than 3. Only Guangzhou, after the neighboring high-risk city of Shenzhen, tightened its home-purchase limit policy, and also increased the strength of its home-purchase limit policy to 6. Once the home-purchase limit policy in some medium-risk cities is strengthened, other cities will make themselves more attractive to talent by increasing their housing supply, which leads to lower housing prices. Another reason is other medium-risk cities still have some limitations on the housing demand of the floating population. These people can move to low-risk cities with weaker home-purchase limit policies to purchase a home or move to high-risk cities with greater opportunities to rent a home. Consequently, demand for housing in other cities has not increased to a significant extent.

According to empirical evidence from the literature, higher policy uncertainty can dampen investment activity [57, 58]. It is reasonable to assume that uncertainty about home-purchase limit policy in medium-risk cities will also have an impact on the housing market, including reduced consumption and irrational volatility [41].

Low-risk cities already have relatively low strength of home-purchase limit policy, So the home-purchase limit policy’s positive spillover effect on housing prices was aggravated in the same type of cities. People can easily flow to other cities of the same type because of the more restrictive policies. Even some cities have not reimplemented the home-purchase limit policy after it was canceled in 2014 or 2015, like Hohhot, Changchun, Harbin, Guiyang, Xining, Yinchuan, and Urumqi. The new demand for housing raises housing prices in other low-risk cities with weaker home-purchase limits.

But the increase in GDP in low-risk cities has a negative spillover effect on housing prices. The possible reason is that the increase in GDP raises the attractiveness of local cities to a certain extent among the same type of cities. When population and housing demand increase in the cities with higher GDP, housing prices in other cities with relatively lower GDP fall due to reduced demand. This is supported by the positive direct effect of the population indicator in low-risk cities.

## Conclusions

This paper uses the spatial Durbin model to examine the spillover effect of home-purchase limit policy on housing prices in 35 large and medium-sized cities in China. We also further divide these cities into high-risk, medium-risk, and low-risk categories to study the heterogeneity of home-purchase limit policy spillover effects.

The main findings are: (1) There is a spatial spillover effect of housing prices in 35 large and medium-sized cities. The values of Moran’s I of housing price coincide with the time when some cities canceled and implemented the home-purchase limit policy. The SDM model is fitted to analyze the spatial spillover effect of housing prices in 35 large and medium-sized cities. (2) In all 35 large and medium-sized cities, the home-purchase limit policy has a significant positive spatial spillover effect on housing prices. (3) The spillover effect of the home-purchase limit policy varies with cities in different types of housing markets. The housing price of those cities with high-risk housing markets are relatively independent and are not impacted by housing price changes in other cities. The spillover effect of the home-purchase limit policy on housing prices is negative in medium-risk cities and positive in low-risk cities, respectively. These findings provide several new insights with respect to the housing policy making.

1. Local governments should formulate the home-purchase limit policy based on the local supply and demand, as well as the strategic objectives of national policies. After the policy is issued, the continuity of the policy should be maintained to avoid fluctuations and spillover in house prices. Especially, high-risk cities, such as Beijing, Shanghai, and Shenzhen, can’t suddenly and frequently change the home purchase limit policy. These cities need to pay more attention to the potential impact on national and regional housing markets when changing their home-purchase limit policy.
2. Local governments should enhance monitoring of local housing markets and other large and medium-sized cities. When other cities tighten their home-purchase limit policy, low-risk cities need to prevent the risk of house price positive spillovers on time. Meanwhile, low-risk cities may also consider improving their competitiveness and attractiveness to create their stable housing market environment.
3. The home-purchase limit policy is an excellent regulatory measure to slow down the growth rate of housing prices. Some medium-risk cities cannot relax housing price regulations because of mutual competition. Instead, these cities should gradually achieve an equal allocation of public resources and services, guide the orderly flow of labor between cities, support efficient industrial upgrading, and improve their ability to regulate the spillovers of housing prices from other cities.
4. The home-purchase limit policy can’t completely solve the housing issues of each large and medium-sized city. Local governments need to combine with other regulatory measures to design more precise and systematic urban housing policy, which is helpful to maintain a healthy and stable development of the housing market.

Of course, because of the complexity and challenge of measuring housing policies, only the home-purchase limit policy is selected for analysis in the paper. Further study may also be conducted for the spillover effect of various housing policy instruments or mixed housing policies. The study on the spillover effects of home-purchase limit policy across time and other heterogeneities also needs to be developed.

## Supporting information

S1 Data Data and weight. (XLSX)
